# Post-stroke hand gesture recognition via one-shot transfer learning using prototypical networks

**DOI:** 10.1186/s12984-024-01398-7

**Published:** 2024-06-12

**Authors:** Hussein Sarwat, Amr Alkhashab, Xinyu Song, Shuo Jiang, Jie Jia, Peter B. Shull

**Affiliations:** 1 School of Mechanical Engineering, Shanghai Jiao Tong University, Dongchuan Road, Shanghai, 200240 China; 2 Robot Offline Programming, Visual Components, Vänrikinkuja, Espoo, 02600 Finland; 3 College of Electronics and Information Engineering, Tongji University, Cao’an Highway, Shanghai, 201804 China; 4 The Department of Rehabilitation Medicine, The National Clinical Research Center for Aging and Medicine, Huashan Hospital, Fudan University, Shanghai, China

**Keywords:** Post-stroke, Hand gesture recognition, Machine learning, Prototypical networks, Few-shot learning

## Abstract

**Background:**

In-home rehabilitation systems are a promising, potential alternative to conventional therapy for stroke survivors. Unfortunately, physiological differences between participants and sensor displacement in wearable sensors pose a significant challenge to classifier performance, particularly for people with stroke who may encounter difficulties repeatedly performing trials. This makes it challenging to create reliable in-home rehabilitation systems that can accurately classify gestures.

**Methods:**

Twenty individuals who suffered a stroke performed seven different gestures (mass flexion, mass extension, wrist volar flexion, wrist dorsiflexion, forearm pronation, forearm supination, and rest) related to activities of daily living. They performed these gestures while wearing EMG sensors on the forearm, as well as FMG sensors and an IMU on the wrist. We developed a model based on prototypical networks for one-shot transfer learning, K-Best feature selection, and increased window size to improve model accuracy. Our model was evaluated against conventional transfer learning with neural networks, as well as subject-dependent and subject-independent classifiers: neural networks, LGBM, LDA, and SVM.

**Results:**

Our proposed model achieved 82.2% hand—gesture classification accuracy, which was better (P<0.05) than one-shot transfer learning with neural networks (63.17%), neural networks (59.72%), LGBM (65.09%), LDA (63.35%), and SVM (54.5%). In addition, our model performed similarly to subject-dependent classifiers, slightly lower than SVM (83.84%) but higher than neural networks (81.62%), LGBM (80.79%), and LDA (74.89%). Using K-Best features improved the accuracy in 3 of the 6 classifiers used for evaluation, while not affecting the accuracy in the other classifiers. Increasing the window size improved the accuracy of all the classifiers by an average of 4.28%.

**Conclusion:**

Our proposed model showed significant improvements in hand—gesture recognition accuracy in individuals who have had a stroke as compared with conventional transfer learning, neural networks and traditional machine learning approaches. In addition, K-Best feature selection and increased window size can further improve the accuracy. This approach could help to alleviate the impact of physiological differences and create a subject-independent model for stroke survivors that improves the classification accuracy of wearable sensors.

*Trial registration number:* The study was registered in Chinese Clinical Trial Registry with registration number CHiCTR1800017568 in 2018/08/04

**Supplementary Information:**

The online version contains supplementary material available at 10.1186/s12984-024-01398-7.

## Background

Stroke is a leading cause of death and disability worldwide [1]. The aging and growing population has caused an increase in the total number of stroke incidents worldwide. While the advances in treatment lowered the mortality rate, the number of survivors in need of rehabilitation has increased substantially [2]. Notably, a significant proportion of such cases is concentrated in lower-income and lower-middle-income countries [3], emphasizing the need for cost-effective interventions that are adaptable across diverse settings.

Stroke rehabilitation is a long, burdensome process, both physically and financially; hence, automated assessment systems that can minimize the rehabilitation costs and reduce the number of visits to physiotherapists are needed [4]. Stroke survivors often exhibit significant variability in their physical conditions, including muscle weakness, spasticity, and altered movement patterns, making it challenging to develop a universal solution. Moreover, the biological signals from stroke survivors differ from those of healthy individuals, further complicating the interpretation of the data [5–7]. These physiological differences underscore the need for tailored sensor designs and analysis techniques capable of accommodating the unique needs and characteristics of stroke survivors. Thus, this study proposes using transfer learning with dimensionality reduction and increased window size to improve the accuracy and applicability of home-based rehabilitation systems.

Recent studies have emphasized the importance of automated assessment and rehabilitation [8, 9]. Automated assessment systems are computerized systems that use sensor data to assess the motor function of stroke survivors. These systems have the potential to replace conventional assessment methods while offering cost-effective means of conducting interactive rehabilitation exercises [10, 11]. This is especially suitable for in-home rehabilitation and can help reduce social isolation [12, 13]. Incorporating games into these systems can motivate post-stroke survivors by enabling them to engage in enjoyable, repetitive motions or tasks [14].

Automated assessment systems typically employ one or more of the following sensors to gather data: electromyography (EMG), force myography (FMG), and inertial measurement units (IMUs). Some systems employ commercially available cameras to get kinematic data [15–17]. However, setting up these cameras requires large space and technical expertise that might not be available in older people. Wearable sensors are easier to set up but have lower accuracy due to differing physiology and inconsistent sensor placement. The work presented in this paper proposes a method that increases the classification accuracy of hand—gestures on new users.

Several studies have investigated different features, dimensionality reduction techniques, and time segmentation on EMG signals [18–21]. However, stroke survivors have intrinsically different biological signals and behavior in contrast to healthier and younger people [22, 23]. Some research suggests that time-domain features of EMG are prone to interference from muscle noise and artifacts. Conversely, other data propose that features in the frequency domain and time-frequency domain exhibit less redundancy concerning the management of nonlinear signal parameters related to muscle spasticity [24].

The use of machine learning for rehabilitation and assessment is becoming increasingly common. Jacob et al. [25] proposed using a deep learning model to extract user intent from electroencephalogram (EEG) signals to stimulate the intended muscle. In the work done by Werner et al. [26], wearable IMUs were employed for assessing participant performance through the utilization of the Action Research Arm Test (ARAT) score. Another study quantified hand and wrist motor function using IMUs and mechanomyography [27]. Li et al. [28] developed a cellphone augmented reality system for long-term treatment of post-stroke patients, which exhibited improvements significantly higher than the control group.

Different studies have investigated the use of gesture recognition for post-stroke rehabilitation. Anastasiev et al. [29] used carefully placed electrodes on forearm muscles to extract EMG signals, reaching an accuracy of 90.37% on new stroke survivors using an SVM classifier. This study was done in a controlled environment where the participant ’s hands and forearms were wiped with alcohol wipes and the muscles were examined by a specialist. Nevertheless, this demonstrates the feasibility of using biological signals for gesture recognition. The use of EMG signals to control a game was investigated by Yang et al. [30]. They tested on 12 stroke survivors and scored an accuracy of 76.1% using an LDA classifier where each participant was trained individually.

Most algorithms operate under the assumption that training and test data originate from the same feature space and exhibit similar distributions [31]. However, this assumption may not always be valid in biological signals, particularly when dealing with electrode shifts or varying user scenarios. Maintaining high performance often necessitates collecting large amounts of data and training a unique model for each user, which is a highly time-consuming and labor-intensive process. Hence, transfer learning can adapt an existing model’s parameters or modify its architecture to suit the new users or tasks, while also reducing the training time and improving the accuracy [32]. Côté-Allard et al. [33] proposed a transfer-learning scheme that uses a source network pre-trained from source-domain, and adding a second network that is trained on the new participant for hand—gesture classification. This transfer learning architecture enhanced the performance on all tested deep learning models. Xu et al. [34] uses their proposed EEGnet, pretrained from source-domain, and fine-tunes the last layer of the network on the new participant. This approach enhances classification accuracy in motor imagery tasks for stroke rehabilitation via brain-computer interfaces. Zhang et al. [35] utilized LSTM neural networks in conjunction with transfer learning to enhance the generalizability of their model across new participants. Zou et al. [36] employed transfer learning to predict knee contact force in participants with knee osteoarthritis. Their findings suggest that transfer learning is simpler and also yields superior results compared to traditional machine learning methods and inverse dynamic analysis.

The research presented in this paper improves the classification accuracy of subject-independent models for hand-gesture recognition post-stroke by employing three distinct methods. The first method significantly improves the performance, and the second and third methods can be applied individually or combined to supplement the first approach. First, using prototypical networks for one-shot transfer learning from the new participant to improve model accuracy from subject-independent models. Second, feature selection and dimensionality reduction are optimized, where applicable, over different classifiers. Third, increased window size to improve model accuracy. To the best of our knowledge, this is the first paper to propose the application of few-shot learning for adapting a generalized model to individual users. The proposed approach is contrasted against conventional transfer learning as well as subject-dependent and subject-independent classifiers and evaluated on data collected from 20 stroke survivors performing seven distinct gestures.

## Methods

The authors acknowledge the use of Language Models (LLMs) for the initial drafting and editing of certain sections of this paper. However, all content has undergone meticulous review and revision by the authors to ensure accuracy, clarity, and adherence to scientific standards.

### Subjects

In this work, we collected data from 20 participants (Table 1) with stroke (Brunnstorm stage for hand 2-6). A medical physician aided in conducting the experiment with all participants. The study was conducted at Huashan Hospital’s Rehabilitation Medicine Department in Shanghai, China. Informed consent was obtained from all participants. The Huashan Hospital Institutional Review Board (CHiCTR1800017568) granted prior approval for the experiment, which was conducted in adherence to the Declaration of Helsinki.

**Table 1:** Participant Information

Sex (M/F)	14/6
Age (mean ± SD)	63.8 ± 14.8
Brunnstrom stage for hand (mean ± SD)	4.5 ± 1.3
FMA upper extremity score (mean ± SD)	42.6 ± 14.2
Hemiplegic side (left/right)	12/8
Diagnosis (ischemic/hemorrhagic)	16/4

### Sensors

A combination of wearable sensors was employed to gather data from the participants. One wristband, with one IMU and eight barometric pressure sensors, was placed on the wrist. The other wristband, with six EMG sensors, was placed on the forearm around 10 cm away from the elbow.

In the first wristband, a 9-axis IMU (BNO055; BOSCH Inc., Stuttgart, Baden-Württemberg, German) was used to gather kinematic data. 3D Eulers angles were also extracted in addition to the data gathered from accelerometers, gyroscopes, and magnetometers [37]. To measure the FMG of tendon sliding, 8 barometric sensors (MPL115A2, Freescale Semiconductor Inc., Austin, TX, United States) were encased in VytaFlex rubber and positioned near the distal end of the ulna on the wrist. The data for both IMU and FMG were collected at 36 Hz and were processed using a 4th-order low-pass Butterworth filter with a cut-off frequency of 5 Hz.

In the second wristband, six wireless EMG sensors from the Trigno Wireless EMG System (MAN-012-2-6, Delsys Inc., Natick, MA, United States) were evenly distributed and placed around the forearm of the participant ’s affected side. The raw EMG data was collected at 1926 Hz and processed using a 4th-order band-pass Butterworth filter with a cut-off frequency of 20 Hz and 500 Hz. The data was then filtered using a Hampel filter to remove artifacts from the data by identifying and removing outliers more than twice the standard deviation away from the average of the surrounding 100 samples.

### Experimental protocol

**Fig. 1:**
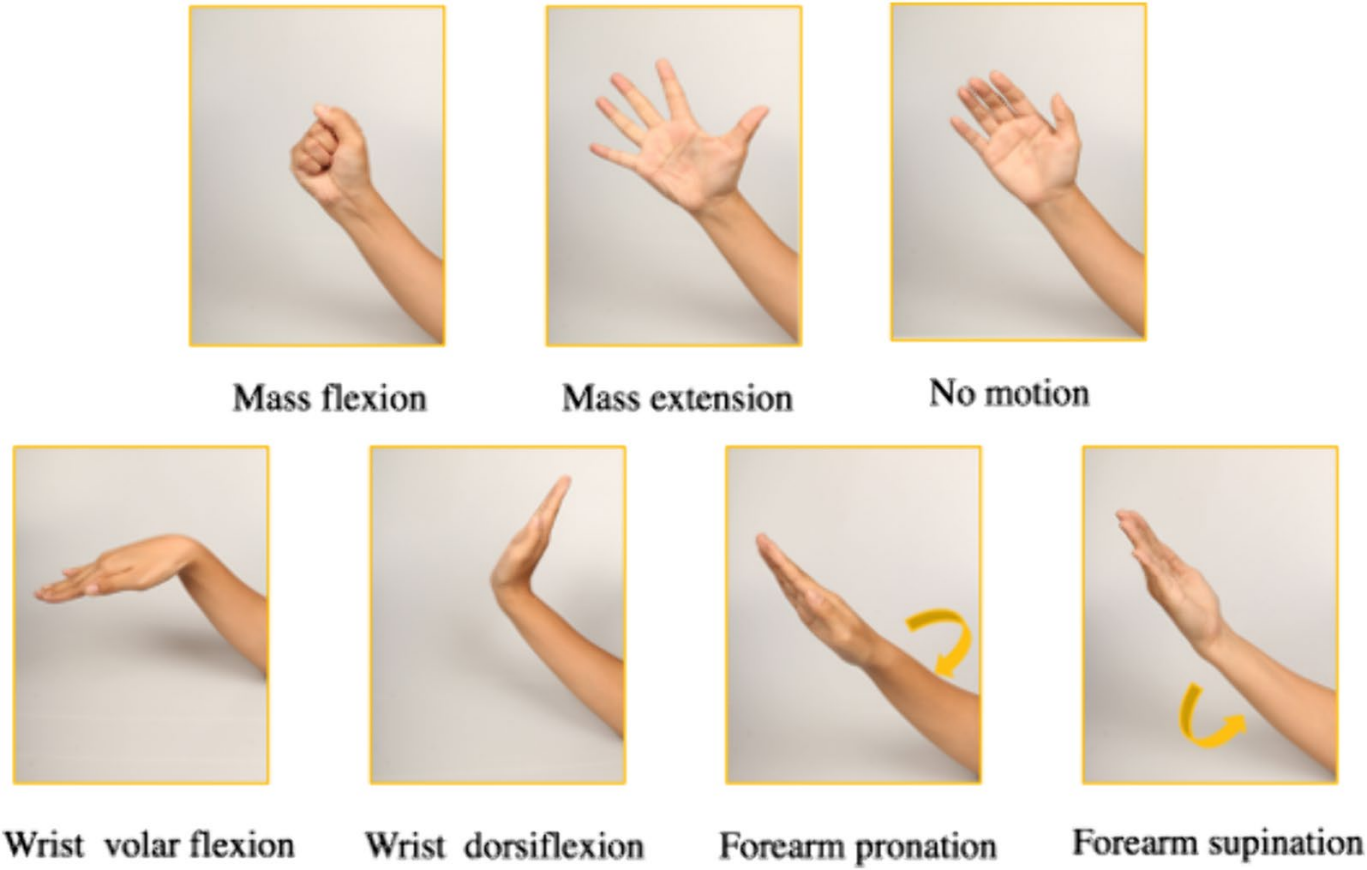
The seven gestures used in the trial

The participants were instructed to sit on a chair with no armrests, allowing their affected arm to hang naturally by their side (shoulder abduction). Before collecting the data, a medical professional explained all the gestures and presented instructional images to the participants. Then, the participants were asked to perform gestures according to the instructional software to familiarize themselves with both the gestures and the software. The software displayed text descriptions and images of the current gesture and the subsequent one. Following this familiarization period, with the assistance of a medical professional, the participants wore the wristbands. Afterward, the participants were instructed to complete five formal trials, with one-minute breaks between each trial. Each trial involved collecting data from seven gestures (Fig. 1), provided in the same order, each gesture lasted 6 s with a 4 s break between each gesture.

### Signal pre-processing and feature extraction

The data from all sensors was collected using MATLAB (MathWorks, Natick, MA, United States) and processed using Python (Python Software Foundation, https://www.python.org/). After filtration, the data was normalized using the mean value and standard deviation from each respective trial. Then the data was segmented using an overlapped segmentation method with a window size of 222 milliseconds and a step size of 55.6 milliseconds. Oskoei and Hu [21] found that an overlapping segmentation approach to EMG data with a window size of 200 milliseconds and a step size of 50 milliseconds provides a quick response time while Junior et al. [20] recommends a step size of 500 milliseconds with a 25% overlap. Both of those studies were tested on healthy participants. Further investigations on window size were done in this study by scaling it up to a factor of 4.

Feature selection is a crucial step in gesture recognition. Effective feature selection enhances classification accuracy, reduces computational complexity, and facilitates the extraction of relevant information from the signals. Thus, from each IMU, FMG, and EMG channel, a total of 12, 14, and 23 features were extracted, respectively, for a total of 394 features. This includes features in the time domain, frequency domain, and time-frequency domain (Table 2).

From the time domain, statistical features such as Mean Absolute Value (MAV), Root-Mean-Square (RMS), Standard deviation (SD), Skew, Kurtosis, and Modified Mean Absolute Value 2 (MMAV2) were extracted. Additionally, Waveform Length (WL), Slope Sign Change (SSC), and Zero Crossing (ZC) were extracted to show the signal’s complexity and frequency information, and reduce noise interference. Other time domain parameters extracted include the Range (RNG), Trapezoidal Integration (INT), Simple Square Integral (SSI), Cardinality (CARD), and 4th and 5th order Temporal Moments (TM4, TM5). CARD is the number of distinct values within a certain threshold (0.001) present in the time-series signal.

Information for the frequency domain was extracted using the Fast-Fourier-Transform. These features are Dominant Frequency (DF), Mean Frequency (MF), Mean Power (MP), and Power Ratio (PR). DF refers to the primary oscillation with the highest amplitude, signifying the most prominent periodic component within the signal. MP provides a representative assessment of the overall energy content, while PR assesses the distribution of power within designated frequency bands expressed as the ratio of power below and above the MF.

Wavelet transform (WT) and Hilbert Huang Transform (HHT) were used for features in the time-frequency domain [38, 39]. WT (’db4’) involves decomposing EMG signals into different frequency components at varying scales, providing a time-frequency representation that captures both temporal and spectral features critical for discriminating distinct muscle activities. The two main components obtained through the decomposition of a signal at different scales or resolutions are ’approximations’ and ’details’. ’Approximations’ refer to the low-frequency components, capturing overall trends, while “details” represent high-frequency components, highlighting rapid changes or fluctuations in the signal. This decomposition enables a hierarchical representation of the signal at different scales, providing a comprehensive view of both coarse and fine details. The HHT is a data analysis method that decomposes a complex signal into intrinsic mode functions (IMF) using empirical mode decomposition and provides a time-frequency representation through the Hilbert spectral analysis. The envelope and the amplitude are extracted through this decomposition, where the envelope represents the upper outline of each IMF, and the amplitude reflects the magnitude or strength of the oscillations associated with each IMF. Using the median for these four features is a better representative for people with stroke than the mean as per Phinyomark et al. [40].

After the features were extracted, the data was normalized again using mean and standard deviation from one participant only. This participant was selected based on the participant with the highest individual accuracy. Normalizing the data using the mean and standard from one participant only had a higher accuracy than normalizing using the mean and standard deviation for all participants, as participants with poor performance or high noise would reduce the accuracy of the results.

**Table 2:** Extracted features from sensors in different domains

Domain	Features
Time Domain	MAV, RMS, SD, Skew^1^, Kurtosis^1^, MMAV2^2^, WL,
	SSC, ZC, RNG, INT, SSI^2^, CARD^2^, TM4^2^, TM5^2^
Frequency Domain	DF, MF, MP, PR
Time-Frequency Domain	Median WT Approximate^2^, Median WT Detail^2^
	Median HHT Envelope^2^, Median HHT Amplitude^2^

Features without a number were extracted from all sensors

^1^Features extracted from FMG and EMG sensors

^2^Features extracted only from EMG sensors

To lower the computational complexity and the processing time, two-dimensionality reduction techniques were assessed on the employed classifiers. These were evaluated by reducing the number of components to 40, and adding 20 till 300 out of the 394 components were used. The first method involves the use of Principal Component Analysis (PCA), which is a widely used statistical technique in data analysis and dimensionality reduction. Its primary goal is to transform a high-dimensional dataset into a lower-dimensional one while retaining as much of the original variability as possible. The second method selects the best k features (K-Best) using the analysis of variance (ANOVA) F-statistic, where k in this case is the number of components.

### Classifiers

**Fig. 2:**
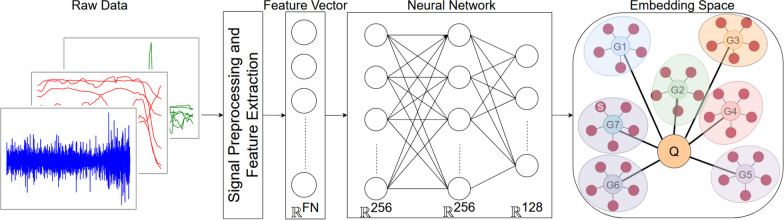
The feature vector FN is fed into a fully connected neural network to generate embedding features. These features map each class prototype (G1, G2,... G7), obtained from the mean of the support set (s), to a position in the embedding space. The class for each new sample (Q) is chosen by using a distance function to identify the closest class prototype

Subject-independent models and models trained using transfer learning were mainly used in this study. Subject-dependent models were used for a final evaluation to compare between the accuracy of general and individual based models. Subject-independent models were trained on all participants with a leave-one-subject out approach, whilst subject-dependent models were trained for each participant individually, with a leave-one-trial out approach. For transfer learning, our proposed model (Fig. 2) using prototypical networks (PN) [41] and neural networks (TL) both used few-shot learning from one to five samples from the new participant’s data. Neural networks (NN), Linear Discriminant Analysis (LDA), Light Gradient Boosting Method (LGBM) [42], and Support Vector Machine (SVM) were employed for subject-independent and subject-dependent models.

Neural networks are composed of interconnected nodes that transmit weighted signals to each other. The input data is processed through three fully connected layers using the ’RelU’ activation function, before passing through a ’Softamx’ activation function to the output layer. This model was trained with a learning rate of 0.0005, a batch size of 20, and 200 epochs. For TL, the model was then trained again using the same parameters on a few samples from the new participant.

Prototypical networks are a type of neural network architecture designed for few-shot learning tasks, they have a query set and a support set. The query set comprises instances for which the model is tasked with making predictions, while the support set includes examples used for creating class prototypes during the training phase (G1, G2,... G7). A prototype is a representative example of a class and is computed as the mean of the embedding of the support set in a given class. The model is trained to classify instances in the query set based on their similarity to these prototypes. This approach enables effective few-shot learning by leveraging a small support set to generalize and make predictions for the new participant.

The training and testing data were divided using a leave-one-subject-out approach. Specifically, during training, only data from the training participants served as the query set, while the support set comprised samples (determined by the number of shots) from the new participant. These samples were taken from different trials, hence a one-shot took one sample from only one of the trials, while a five-shot took one sample from each of the trials. Subsequently, during testing, the same set of samples was employed as the support set, whereas the remaining samples from the participant were utilized to assess the performance of the trained model.

During training, a prototypical network processes the support set through a shared neural network to generate embeddings. The prototypes for each class are then computed as the mean of these embeddings for all examples in the support set that belong to the same class. The query set is similarly embedded using the same neural network. The similarity between the embeddings of the query set and the class prototypes is calculated using Euclidean distance as a metric. The softmax function applied to the negative of these distances yields a probability distribution over the classes, where a shorter distance corresponds to a higher probability of class membership. The loss function is calculated as the negative log likelihood of the true class label, based on these probabilities. This loss is then used to update the weights of the neural network through backpropagation.

Once the prototypes have been computed, the classification process involves comparing new data points to these prototypes in the embedding space and assigning them to the class with the closest match. This approach is effective in few-shot learning tasks because it captures the essence of each class with a limited number of examples. The distance metric used to measure the similarity between a data point and a prototype is the Euclidean distance:

1 $$\begin{aligned} d(x,p) = ||x-p||^2 \end{aligned}$$

where $||x-p||$ is the Euclidean norm of the difference between vector *x* and *p*. Afterwards, the class assignment is determined using

2 $$\begin{aligned} y = argmin_g d(x, p_g) \end{aligned}$$

where *y* is the predicted gesture for *x*, *g* is the gesture index, and $p_g$ is the prototype for gesture *c*. Several different classifiers were used to evaluate the performance of the proposed method.

SVMs are particularly well-suited for high-dimensional data and are known for their generalization ability and robustness to noise, making them suitable for the current problem and have been used in similar studies [29, 43]. A one-vs-one decision function with an ’rbf’ kernel with a kernel coefficient $\gamma =\frac{1}{FN}$, where *FN* is the number of features, and a regularization parameter $C=1$ and a were used in this study. LGBM is a powerful gradient-boosting framework that employs decision trees as weak learners to construct a robust ensemble model. LGBM generally performs better than Decision Trees and Random Forests and has been used by Formstone et al. [27] for quantification of motor function. A multi-class one-vs-all configuration with 300 boosted trees was used in this study. LDA is a statistical method that finds a linear combination of features that best distinguishes between two or more classes of data. It can also reduce training time while still maintaining accuracy [44], making it a good option for real-time gesture recognition [30, 45].

### Statistical analysis

For subject-independent models, each approach was repeated 20 times, where a different participant was left out or used for transfer learning, depending on the approach. For subject-dependent models, each approach was repeated 5 times per participant, where a different trial was left out. The average of the five trials for each participant was recorded, and the mean of all the participants was used to determine the accuracy of the classifier. A one-way ANOVA was employed to calculate the statistical significance between different approaches and techniques. The Benjamini-Hochberg method to control the false discovery rate was used to adjust all of the computed p-values [46]. Any of the adjusted p-values lower than 0.05 was considered statistically significant.

## Results

**Fig. 3:**
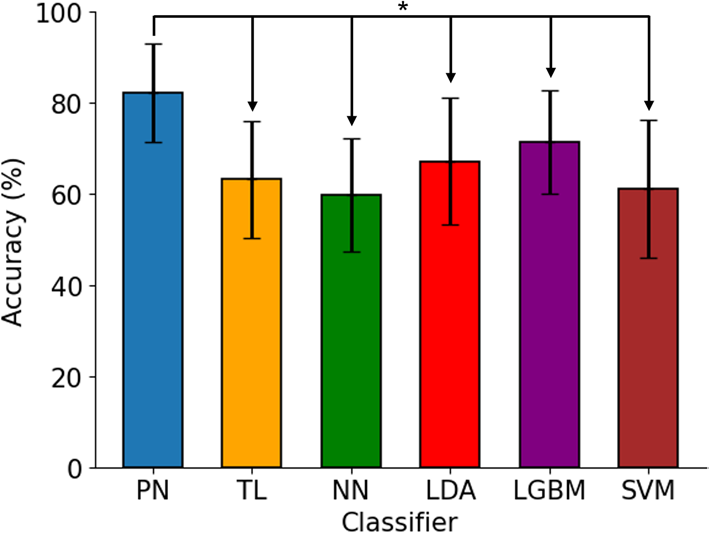
Our proposed methodology (one-shot PN with 0.88s window size) was significantly more accurate than all other benchmark models ($p<0.05$)

**Fig. 4:**
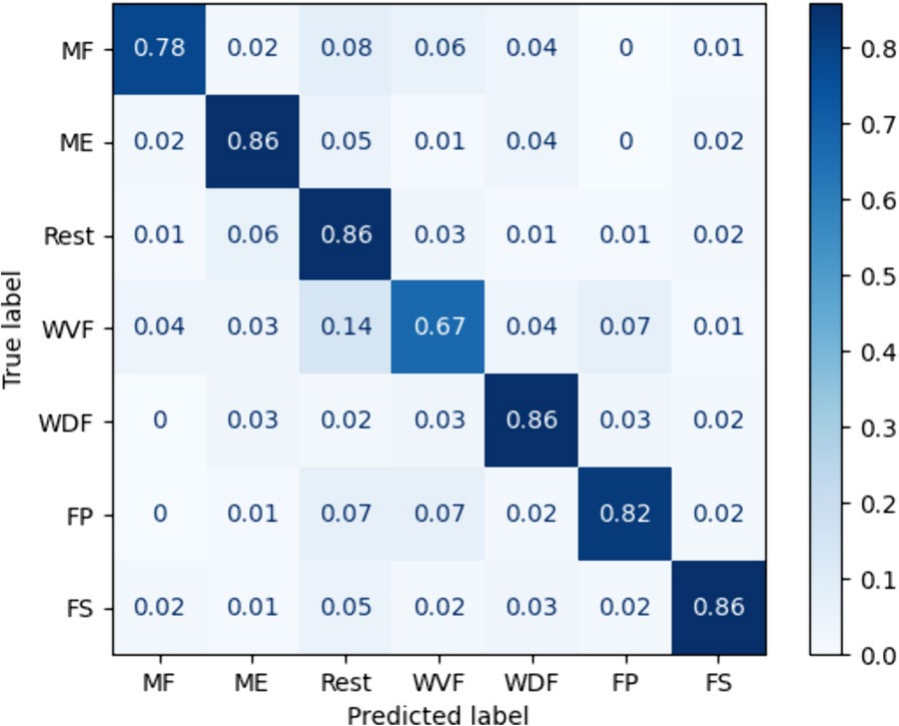
Confusion matrix for our proposed one-shot approach

Using a one-shot approach with a large window size significantly improved the accuracy ($p<0.05$) in comparison to TL and other subject-independent classifiers (Fig. 3). Our approach scored an accuracy of $82.20\% \pm 10.85\%$, significantly higher than all other subject-independent classifiers, followed by LGBM with an accuracy of $71.43\% \pm 11.35\%$. The confusion matrix (Fig. 4) presents the classification accuracy for each gesture. The one shot approach was evaluated against other optimised classifiers with larger window size and K-Best features, where applicable. Further evaluation of PN and TL used the five-shot approach with the smaller window size for the larger number of samples.

### Feature selection and dimensionality reduction

**Fig. 5:**
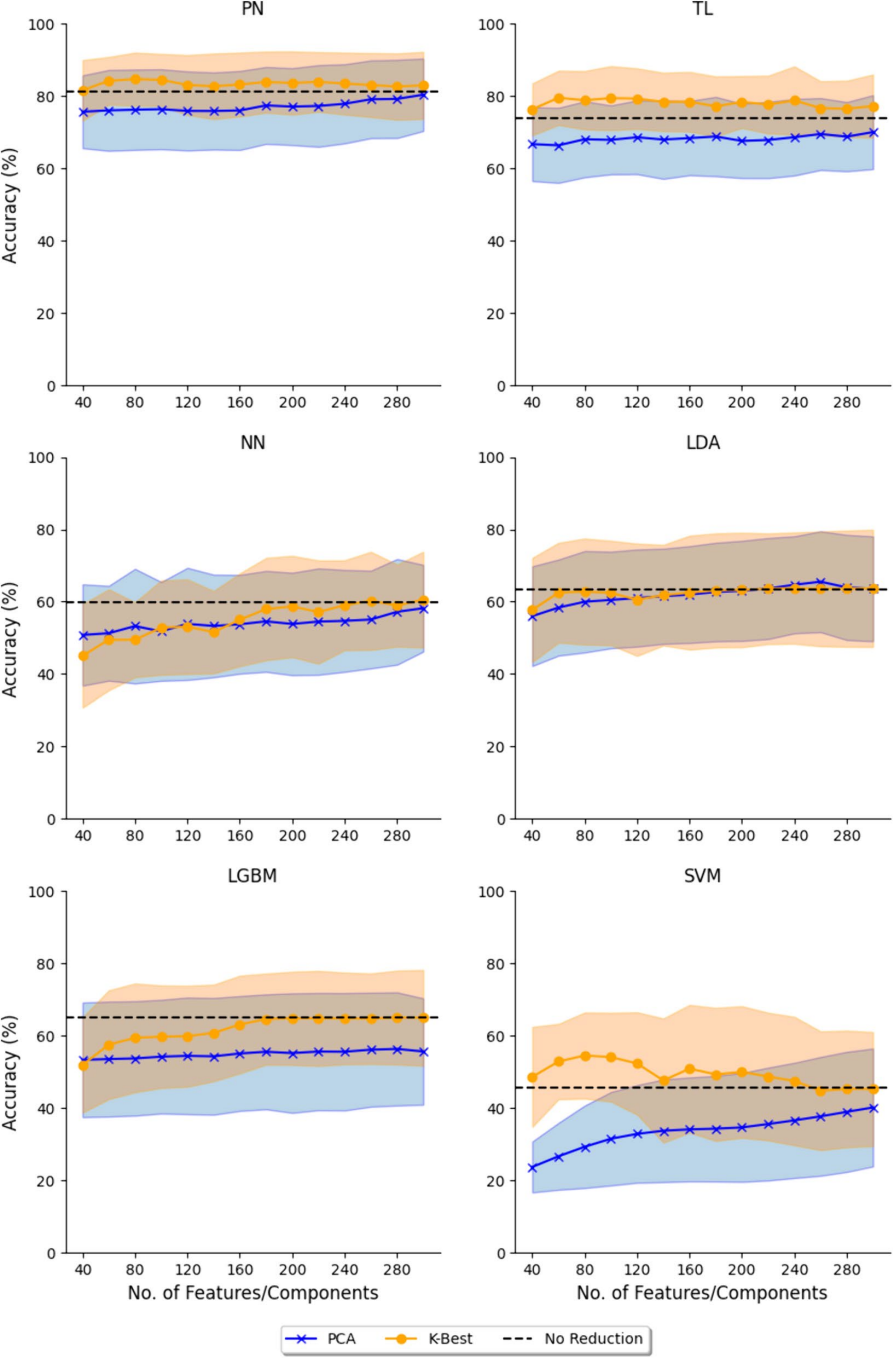
Effect of dimensionality reduction using PCA and K-Best features selection. The shaded area represents the standard deviation. Five-shot PN and TL, and SVM have better accuracy at lower features, while LDA is not affected. NN and LGBM have lower accuracy with features, but plateau at higher number of features

Each classifier responded differently to using K-Best or PCA for dimensionality reduction (Fig. 5). Dimensionality reduction using K-Best features enhanced the performance of PN, TL, and SVM, attaining peak accuracies at 80, 60, and 80 features, respectively. This led to accuracy improvements of 3.57%, 5.55%, and 9.02%, respectively. On the other hand, the performance of NN and LGBM improved with increasing the number of features till they plateaued at 300 and 280 features, where the difference was negligible. LDA demonstrated the least sensitivity to dimensionality reduction. The highest accuracy was observed when employing PCA with 260 components, resulting in an accuracy of 65.53%, resulting in a 2.18% increase compared to no reduction. PN and TN were evaluated with a five-shot approach and a small window size for a larger sample size.

**Fig. 6:**
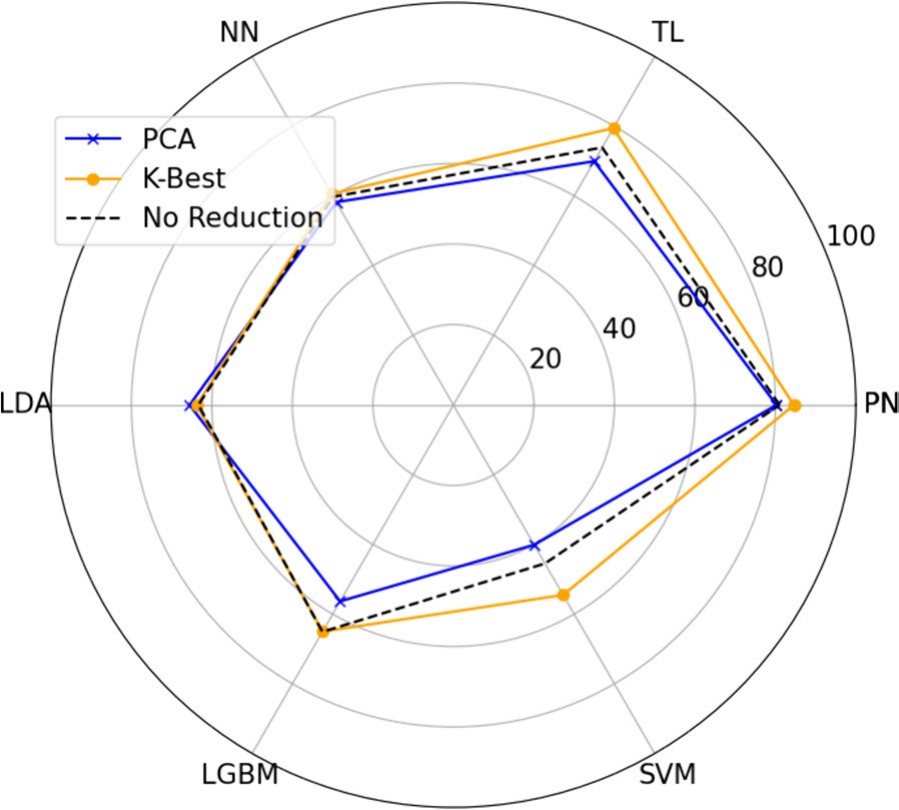
Comparison between best accuracy achieved when using PCA or K-Best and without reduction

Across the six different utilized classifiers, PCA demonstrated the worst performance with only LDA having a higher accuracy when utilizing PCA. The discrepancy between employing K-Best and not was minimal, with a difference of less than 1%. Conversely, for the remaining classifiers, utilizing K-Best resulted in the most optimal performance. Figure 6 compares between the optimal results (extracted from Fig. 5) for different classifers and different dimensionality reduction.

### Window size

For evaluating different window sizes, NN, LDA, and LGBM classifiers were utilized without dimensionality reduction due to its negligible effect. PN, TL, and SVM classifiers were employed with their optimum dimensionality reduction configuration, and PN and TN were evaluated with a five-shot approach.

**Fig. 7:**
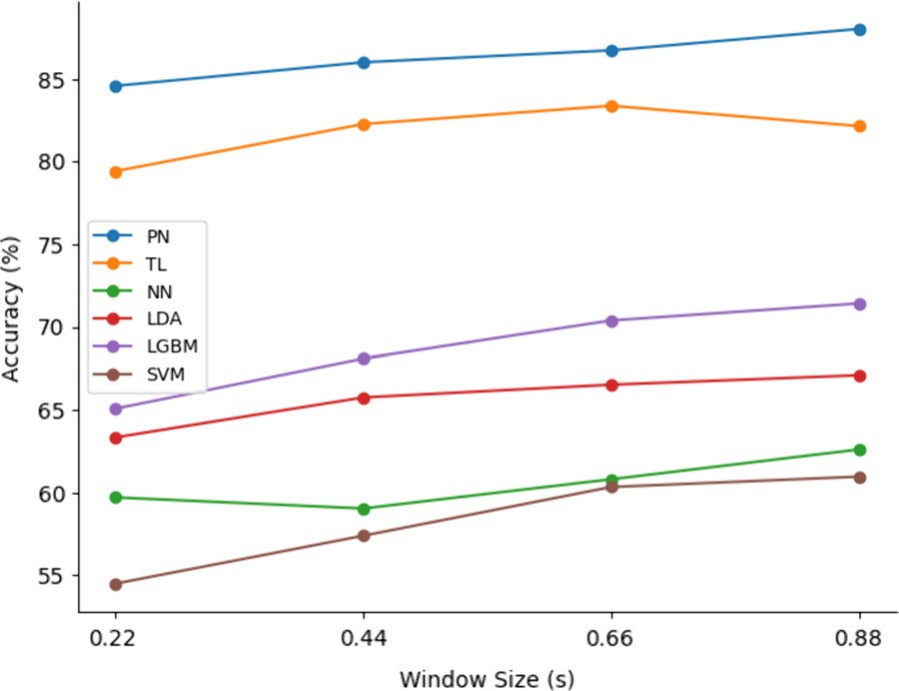
Effect of window size on different classifiers. Increasing the window size slightly improved the accuracy over all the classifiers

Enlarging the window size resulted in enhanced accuracy across all classifiers, despite the reduced sample size (Fig. 7) . The most notable improvements were observed in SVM and LGBM, with accuracy enhancements of 6.48% and 6.34%, respectively. Conversely, NN and TL exhibited comparatively modest accuracy gains, registering improvements of 2.9% and 2.73%, respectively. On average, all classifiers manifested an accuracy augmentation of 4.28%.

### Number of shots

**Fig. 8:**
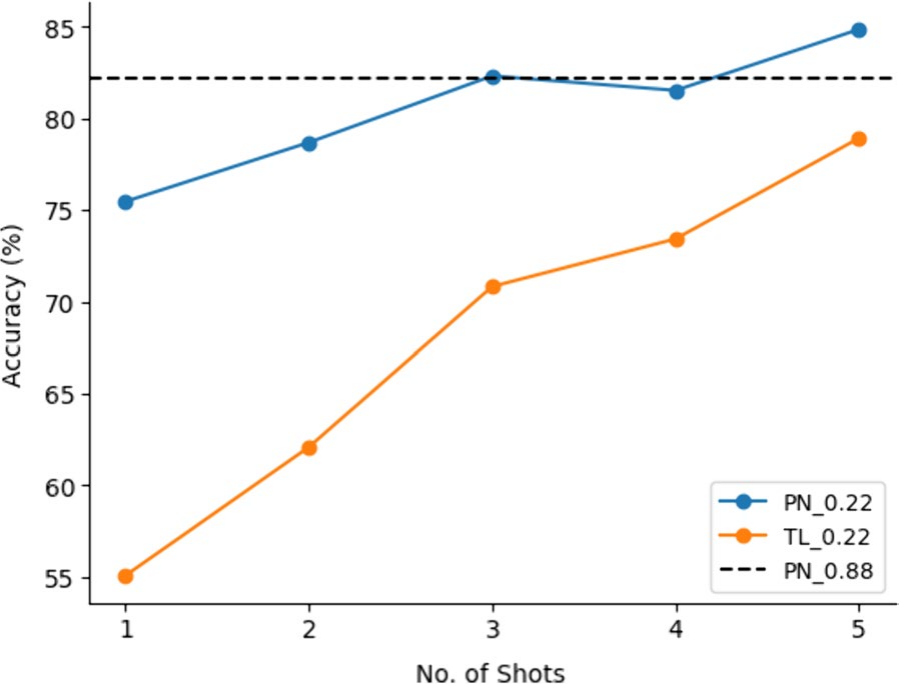
Effect of using more samples from the new participant, with smaller window size, to train the model in contrast to using a one-shot approach with a large window size

Due to overlapping windows, one-shot from the largest window size was compared with up to five-shots from the smallest window size (Fig. 8).

Using a larger window size demonstrated better performance with fewer samples. The smaller window size achieved better accuracy after three samples.

**Fig. 9:**
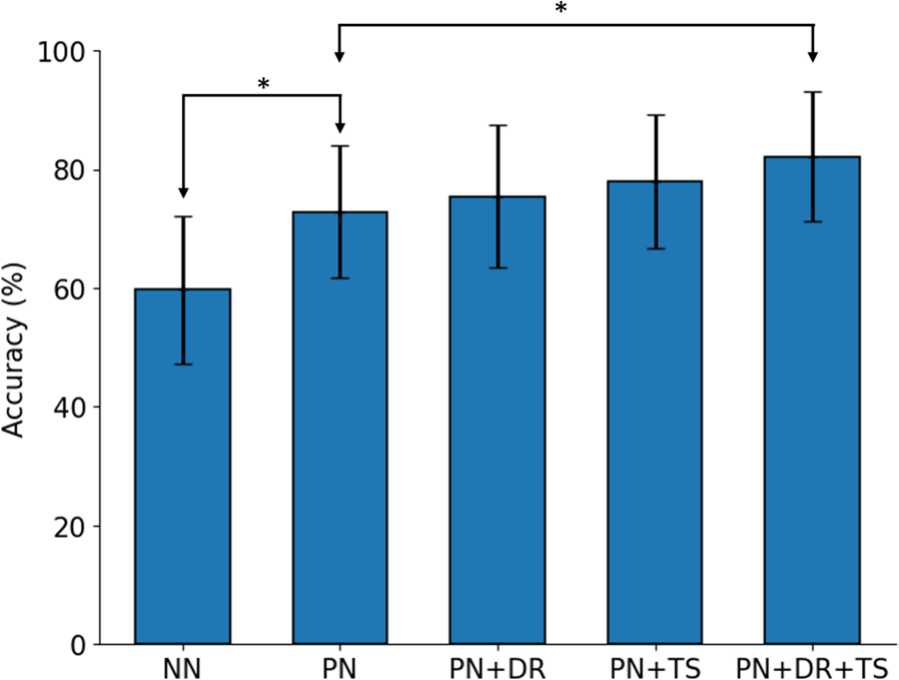
Using one-shot transfer learning (PN) improved the accuracy in comparison to subject-independent NN. Supplementing it with dimensionality reduction (PN+DR), or increased window size (PN+TS), or both (PN+DR+TS) improved the accuracy even further. Results with (*) indicate a significant difference ($p<0.05$) between them

Each of the proposed methods improved the accuracy of the subject-independent model (Fig. 9).

### Subject-dependent

**Fig. 10:**
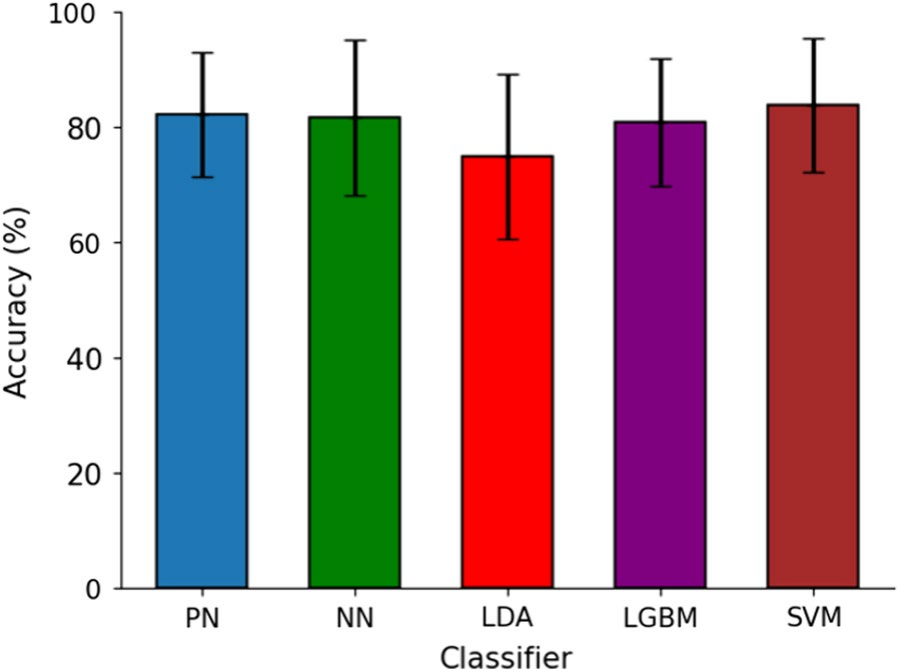
Our proposed approach performed similarly to subject-dependent models

When compared to other subject-dependent classifiers (Fig. 10), our approach demonstrated similar performance, underscoring the robustness of our method in effectively integrating large-scale models into new participants. These classifiers were trained on a large window size and the SVM classifier used K-Best feature selection for dimensionality reduction. SVM demonstrated the highest performance with a $83.84\% \pm 11.65\%$ accuracy, followed by our approach. LDA had the lowest accuracy at $74.89\% \pm 14.36\%$ with at least a $5.9\%$ difference between it and other classifiers. LGBM and NN scored an accuracy of $80.79\% \pm 11.07\%$ and $81.62\% \pm 13.61\%$, respectively.

## Discussion

Current in-home rehabilitation systems lack the reliability of rehabilitation with a physiotherapist. The reason for this is the difficulty of translating the rehabilitation systems to home environments. This can be due to multiple reasons, such as lack of space, lack of technical expertise by the patient, and inability to accurately wear the sensor. The sensor displacement can significantly impact the quality of the data and the performance of the wearable device [47]. To improve the accuracy of wearable systems, we propose the usage of PN to tune previously trained models onto the new user. This form of transfer learning requires only one shot to achieve significantly better accuracy ($p<0.05$) and can reduce the effect of sensor displacement.

Real-time gesture recognition models require short window size for data analysis. While this approach works well for healthy individuals, it may not be suitable for stroke survivors due to the differences in their biological signals. Stroke survivors exhibit more noise, necessitating robust features that are not typically found in the time domain. To address this issue, increasing the time segment can reduce noise and improve gesture recognition performance, even with a smaller sample size. However, this trade-off comes at the expense of the real-time capabilities of the utilized model. While the statistical significance of the results may not have been pronounced, it is notable that a discernible trend emerged across the classifiers utilized in the study. Specifically, a consistent increase in accuracy can be observed as the window size increases. Current literature regarding window size segmentation have found that increasing the window size improves the accuracy up to a certain threshold [20, 21]. Considering the current literature regarding the noisy nature of biological signals emitted from stroke survivors, this threshold might be larger for them. Despite the lack of statistical significance, all these points indicate that there may indeed be meaningful patterns to be uncovered with larger datasets or alternative methodologies.

Feature selection poses a challenge in developing gesture recognition models for stroke survivors. Extracting numerous features, some of which are multidimensional, can significantly increase the parameters of the feature vector, potentially from a few dozen to hundreds or even more, depending on the number of channels. This abundance of features may, depending on the chosen classifier, lead to reduced model performance and increased computational time. Additionally, some of the extracted features may be noisy and decrease the performance of the model. Thus, using K-Best feature selection to eliminate those features usually results in better accuracy than using PCA, which tries to retain as much information as possible.

Different models exhibit varying performance depending on the number of available samples. While healthy users can perform multiple trials to optimize sensor performance, this may be challenging for people with stroke. Utilizing prototypical networks for few-shot transfer learning can substantially enhance model accuracy. The samples gathered for the few-shot learning were disjoint and from different trials. Consequently, despite the better accuracy of the five-shot PN with a small window size, the one-shot PN with a large window size demonstrates greater reliability to unseen data. The five-shot approach will likely have samples from multiple trials, whilst a one-shot approach will only have one sample from one trial.

Time segmentation, dimensionality reduction, and feature extraction techniques have all been investigated for healthy users. This does not translate to people with stroke, as seen in the results displayed in this work. Further analysis and investigations must be conducted to determine the optimal configurations for assessing people with stroke.

A major problem in rehabilitation research is the lack of generalized models that can work well for different people. Current methods often struggle to adapt to the unique conditions of each person. Without these models, it’s hard to successfully implement wearable systems for in-home rehabilitation. Our research shows that when compared to subject-dependent classifiers, our approach consistently achieves similar results. This suggests that our method can effectively use large-scale models in new users without losing accuracy. This flexibility is valuable for rehabilitation research, where subject-specific data are limited. However, it’s important to note that in our study, data was presented in a consistent order to accommodate the slower reaction times of stroke recovery patients, which might limit the generalizability of our findings due to potential learning effects.

This paper proposes the use of prototypical networks for one-shot transfer learning to quickly adapt to new users. This method can greatly improve the performance of wearable sensors for rehabilitation systems, where constant supervision is not possible.

## Conclusion

This paper proposes using prototypical networks for few-shot transfer learning to swiftly adapt to new users. This approach can significantly enhance the performance of wearable sensors in rehabilitation systems and serious games. Additionally, the role of time segmentation and feature selection has been examined to evaluate their significance. Extending the time segment will likely improve performance but compromise real-time capabilities. Feature selection can either improve or degrade model performance, depending on the classifier. Therefore, it is crucial to consider dimensionality reduction techniques that preserve essential information, while removing noisy ones, before feeding the data into the model.

### Supplementary Information

Supplementary Material 1. The code supporting the findings of this study is available in the GitHub repository at https://github.com/HSarwat/Few-Shot-Proto-TL.git.

## Data Availability

Data sets generated during the current study are available from the corresponding author on reasonable request.

## Abbreviations

ADLs: Activities of daily living
ANOVA: Analysis of variance
ARAT: Action research arm test
CARD: Cardinality
DF: Dominant frequency
EEG: Electroencephalogram
EMG: Electromyography
FMG: Force myography
FMA: Fugl-meyer assessment
HHT: Hilbert Huang transform
IMF: Intrinsic mode functions
IMU: Inertial measurement unit
LDA: Linear discriminant analysis
LGBM: Light gradient boosting method
MAV: Mean absolute value
MF: Mean frequency
MP: Mean power
MMAV2: Modified mean absolute value 2
NN: Neural networks
PCA: Principal component analysis
PN: Prototypical networks
PSR: Power ratio
RMS: Root-mean-square
RNG: Range
SD: Standard deviation
SSI: Simple square integral
SSC: Slope sign change
SVM: Support vector machine
TM4: 4th order temporal moment
TM5: 5th order temporal moment
TL: Transfer learning with neural networks
WT: Wavelet transform
WL: Waveform length
ZC: Zero crossing
