# Supplementary material for: Post-stroke hand gesture recognition via one-shot transfer learning using prototypical networks
Source: J Neuroeng Rehabil. 2024 Jun 12;21:100. doi: 10.1186/s12984-024-01398-7 (PMC11167772; doi:10.1186/s12984-024-01398-7)
Supplement: Supplementary file 1 — Supplementary Material 1. The code supporting the findings of this study is available in the GitHub repository at https://github.com/HSarwat/Few-Shot-Proto-TL.git. [file 12984_2024_1398_MOESM1_ESM.pdf]

Supplementary File

This file contains the results for all the classifiers.

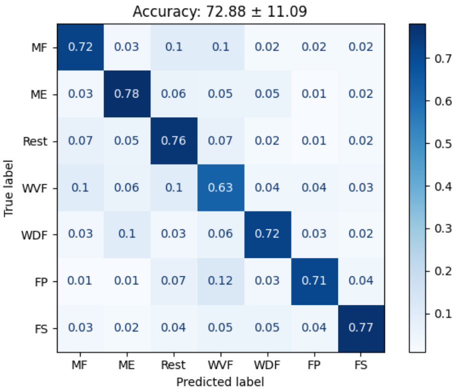

(a) Original

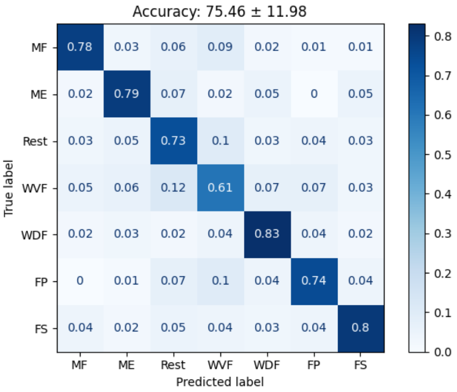

(b) Dimensionality Reduction

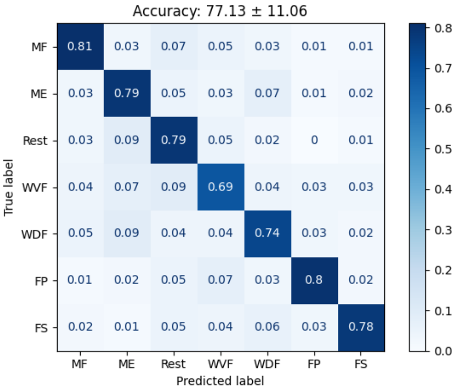

(c) Increased Window Size

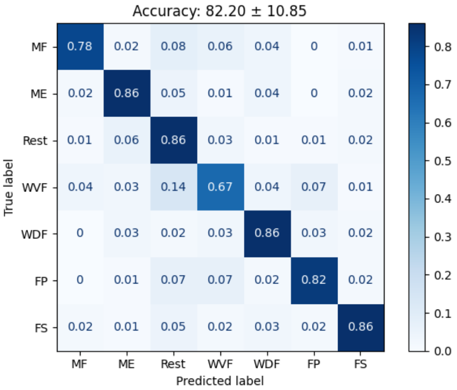

(d) Both

Figure 1: Prototypical Networks

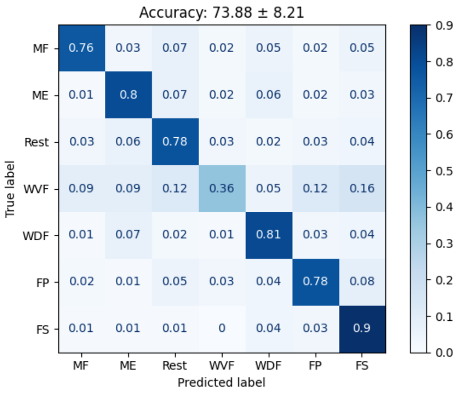

(a) Original

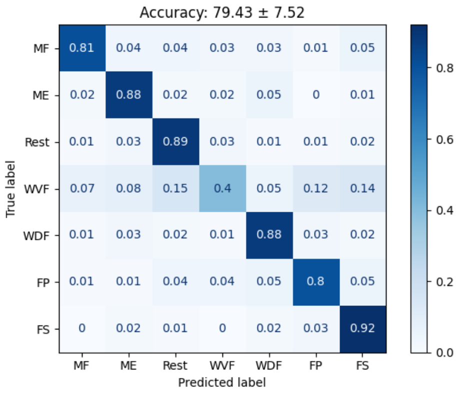

(b) Dimensionality Reduction

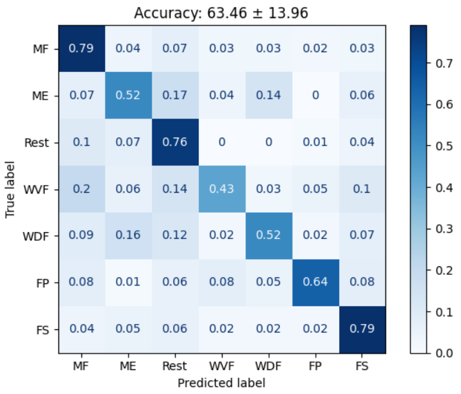

(c) Increased Window Size

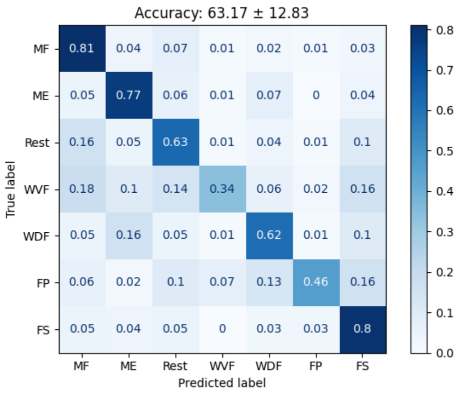

(d) Both

Figure 2: Transfer Learning

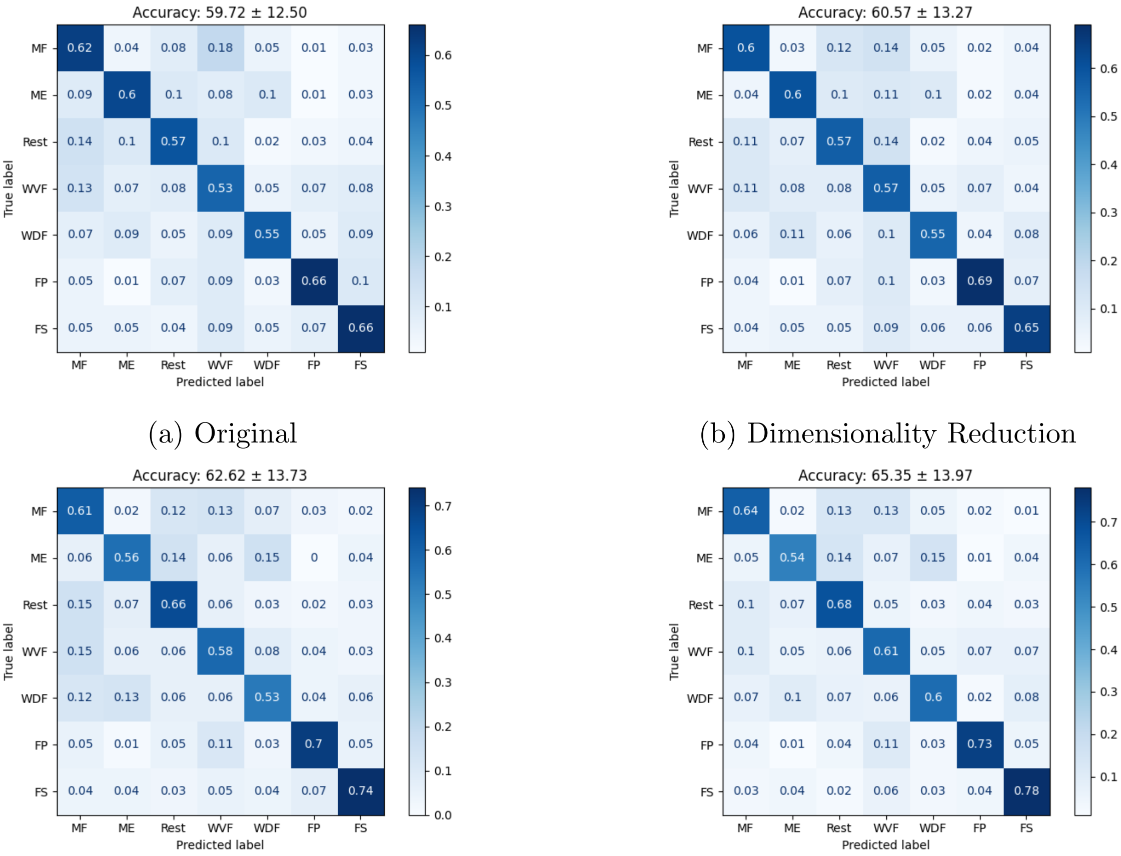

(a) Original

(b) Dimensionality Reduction

(c) Increased Window Size

(d) Both

Figure 3: Neural Networks

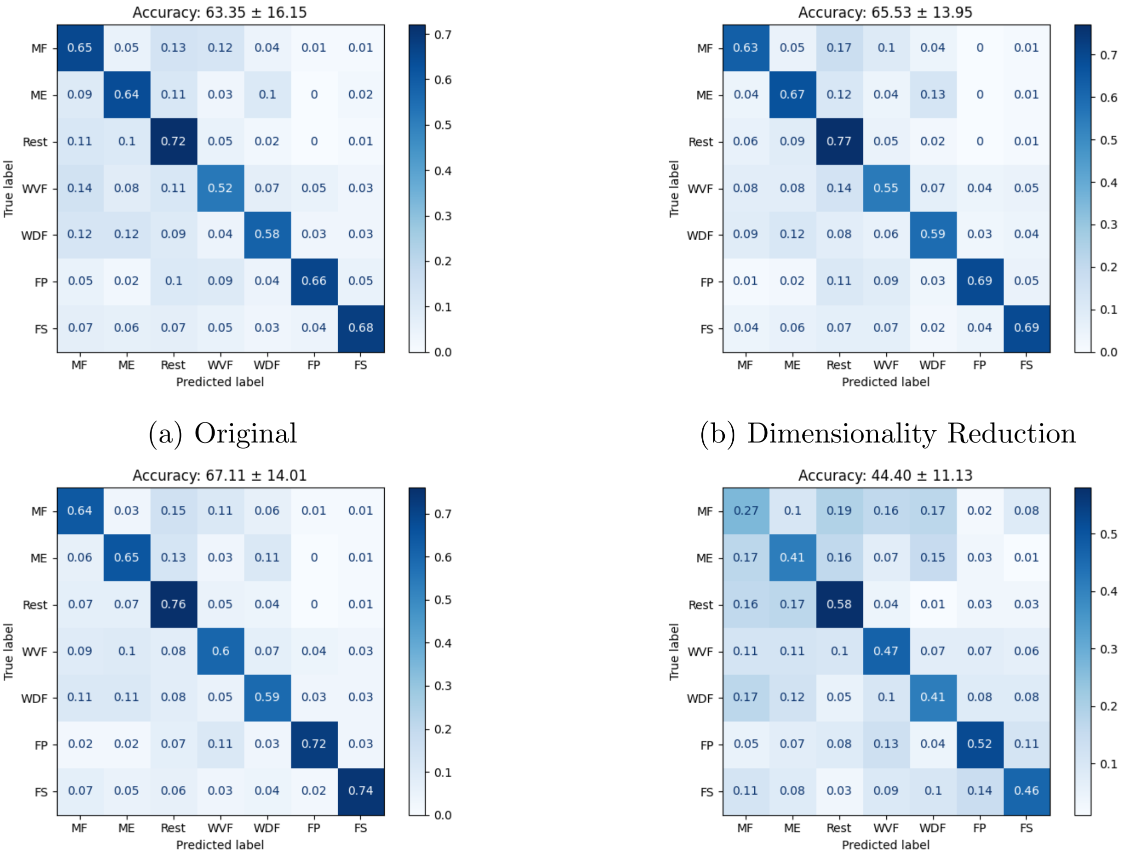

(a) Original

(b) Dimensionality Reduction

(c) Increased Window Size

(d) Both

Figure 4: Linear Discriminant Analysis

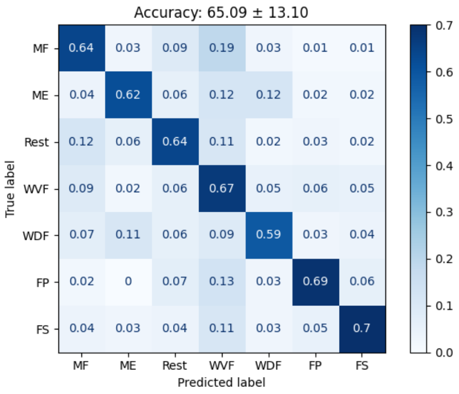

(a) Original

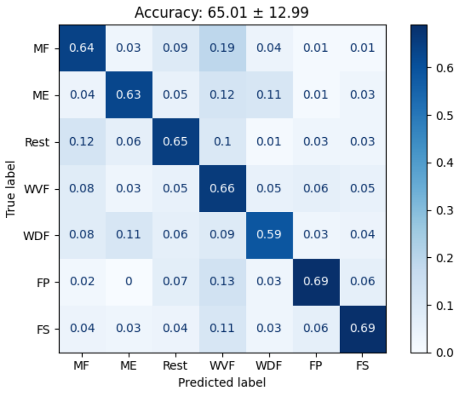

(b) Dimensionality Reduction

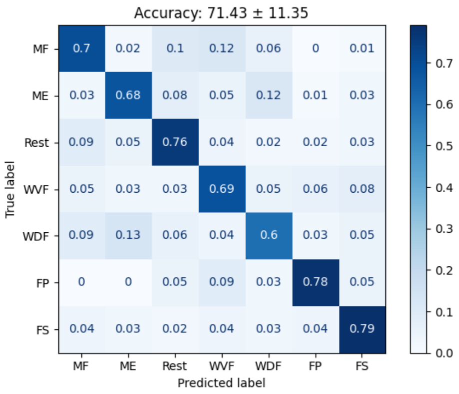

(c) Increased Window Size

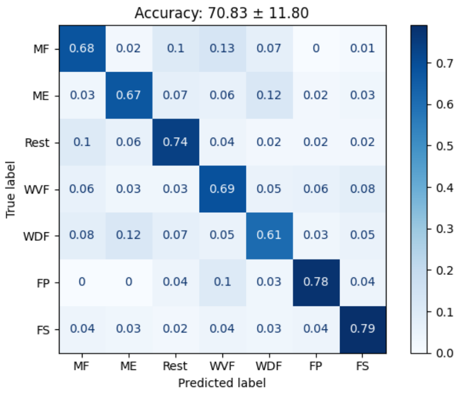

(d) Both

Figure 5: Light Gradient Boosting

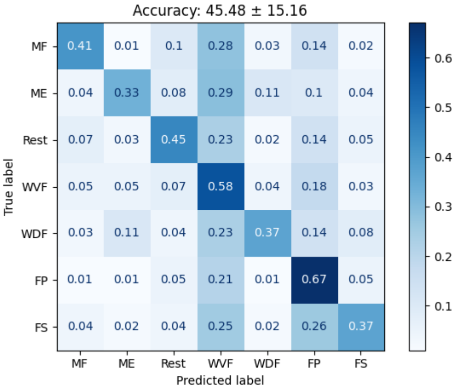

(a) Original

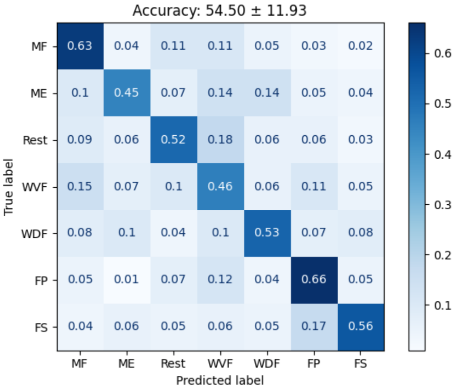

(b) Dimensionality Reduction

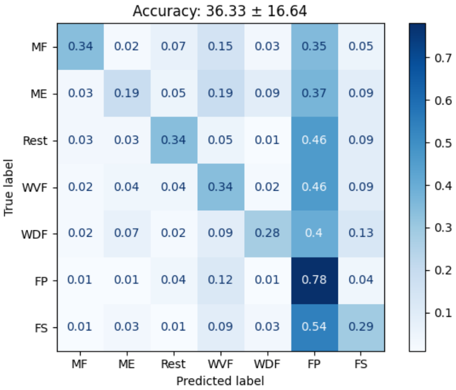

(c) Increased Window Size

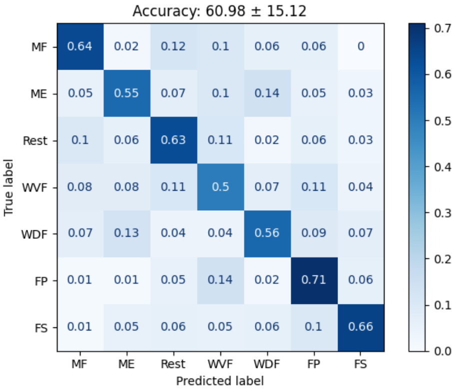

(d) Both

Figure 6: Support Vector Machine
